# Risk factors in developing amyloid related imaging abnormalities (ARIA) and clinical implications

**DOI:** 10.3389/fnins.2024.1326784

**Published:** 2024-01-19

**Authors:** Sarah J. Doran, Russell P. Sawyer

**Affiliations:** Department of Neurology and Rehabilitation Medicine, University of Cincinnati College of Medicine|UC Health, Cincinnati, OH, United States

**Keywords:** ARIA-E, ARIA-H, Alzheimer’s disease, anti-amyloid, Apoε4

## Abstract

Alzheimer’s disease (AD) affects over 6 million people over the age of 65. The advent of new anti-amyloid monoclonal antibodies as treatment for early Alzheimer’s disease these immunotherapeutics may slow disease progression but also pose significant risks. Amyloid related imaging abnormalities (ARIA) identified on MRI following administration of these new monoclonal antibodies can cause both brain edema (ARIA-E) and hemorrhage (ARIA-H). While most ARIA is asymptomatic, some patients can develop headache, confusion, nausea, dizziness, seizures and in rare cases death. By analyzing lecanemab, aducanumab, gantenerumab, donanemab, and bapineuzumab clinical trials; risk factors for developing ARIA can be identified to mitigate some of the ARIA risk. Risk factors for developing ARIA-E are a positive Apoε4 carrier status and prior multiple cerebral microhemorrhages. Risk factors for ARIA-H are age, antithrombotic use, and history of prior strokes. With lecanemab, ARIA-E and ARIA-H were seen at lower rates 12 and 17%, respectively, compared to aducanumab (ARIA-E 35% and ARIA-H 19%) in treated patients. ARIA risk factors have impacted inclusion and exclusion criteria, determining who can receive lecanemab. In some clinics, almost 90% of Alzheimer’s patients are excluded from receiving these new anti-amyloid therapeutics. This review aims to discuss risk factors of ARIA and highlight important areas for further research. With more anti-amyloid monoclonal antibodies approved by the Food and Drug Administration, considering patient risk factors for developing ARIA is important to identify to minimize patient’s risk while receiving these new therapies.

## Introduction

Alzheimer’s disease effects an estimated 6.5 million Americans over 65 years old (Author, 2022). With new anti-amyloid monoclonal antibody therapeutics, there is a possibility of slowing disease progression. However, this is not without risk as these new anti-amyloid therapeutics have been associated with abnormal MRI findings called Amyloid related imaging abnormalities (ARIA). While usually asymptomatic, ARIA can sometimes lead to confusion, encephalopathy, seizures and in very rare cases death (Barakos et al., 2022; Jeong et al., 2022; Reish et al., 2023; Sims et al., 2023). The risk of developing ARIA has been associated with drug dosage, age, microhemorrhages, Apo ε4 allele, history of prior strokes and antithrombotic use (Barakos et al., 2022; Filippi et al., 2022), and these risk factors have impacted FDA inclusion and exclusion guidelines in administration of lecanemab (Supplementary Table S1). Understanding these risk factors as these anti-amyloid therapies become more widely available is an important consideration prior to initiating therapy.

## What is ARIA?

**Figure 1:**
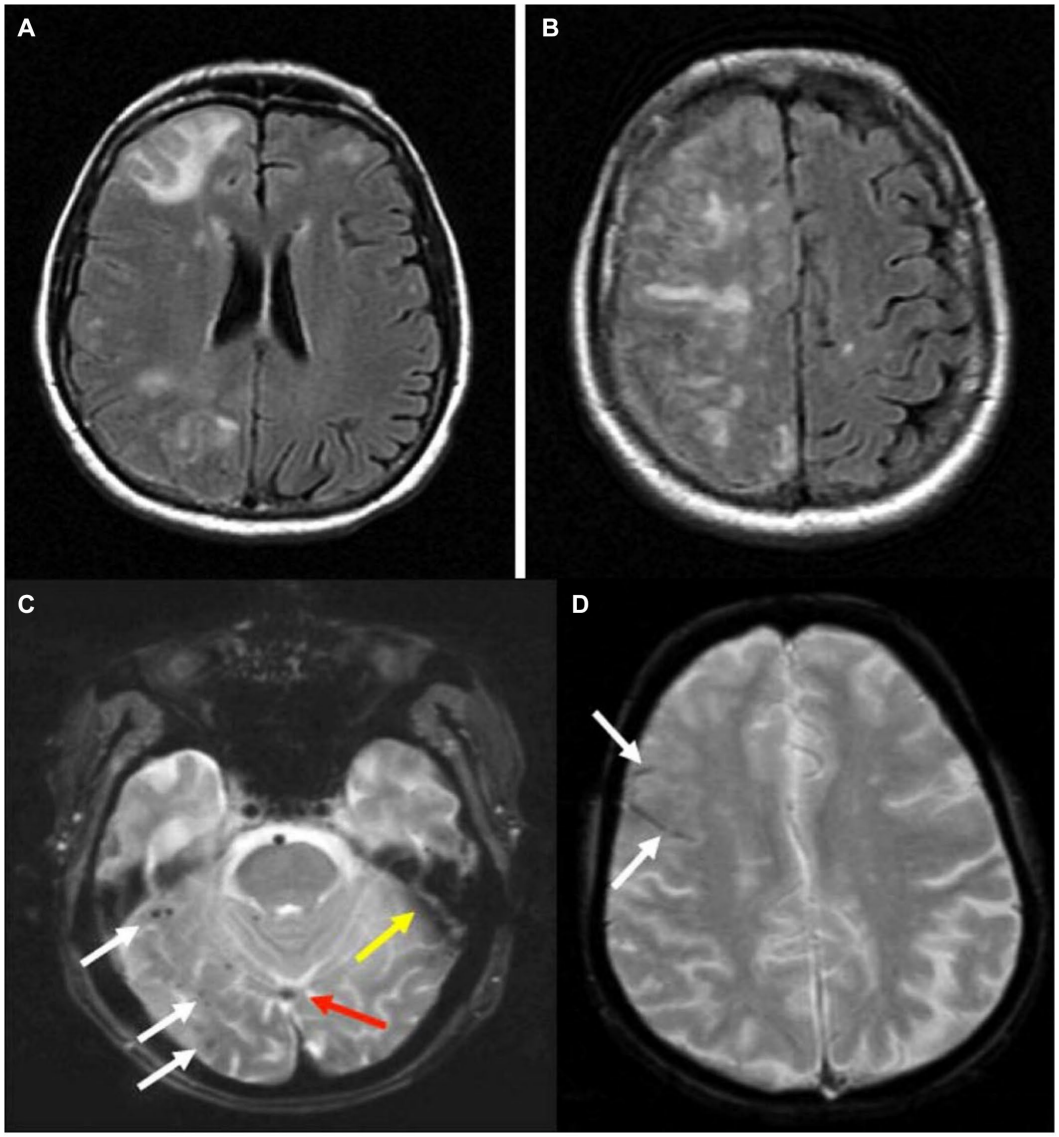
**(A,B)** ARIA-E which occurred during a monoclonal antibody trial, as seen on fluid attenuation inversion recovery (FLAIR) magnetic resonance images demonstrating increased signal in multiple regions of the right hemisphere affecting both gray and white matter. **(C)** Microhemorrhage (mH) and superficial siderosis. **(C)** White arrows indicate multiple 1-to 3-mm dark foci in the right inferior temporal and occipital lobes, typical of the appearance of mH. Red arrow indicates inferior sagittal sinus, and yellow arrow indicates susceptibility artifact, because vascular structures and artifacts can sometimes mimic the appearance of mH and siderosis. **(D)** White arrows indicate curvilinear dark sulci in the right frontal lobe, typical of the appearance of superficial siderosis. Both images were acquired at 1.5 T with a two-dimensional long TE (30 ms) GRE sequence.

ARIA is a term developed in 2011 by the Alzheimer Association Research Roundtable work-group to identify and classify MRI findings in association with anti-amyloid monoclonal therapeutics (Sperling et al., 2011, 2012). ARIA can be divided into two distinct imaging findings associated with different risk factors. The first is amyloid related imaging associated edema or effusion (ARIA-E) is associated with parenchymal edema or sulcal effacement associated with hyperintensities on MRI in T2 weighted imaging or Fluid attenuated inversion recovery (FLAIR) imaging with no associated diffusion restriction on diffusion weighted imaging (Agarwal et al., 2023; Roytman et al., 2023) [Figures 1A,B reproduced from Sperling et al., 2011 with permission from Wiley] (Sperling et al., 2011). While ARIA-E is seen unilaterally in two thirds of cases, it can be seen bilaterally (Barakos et al., 2013). Amyloid related imaging associated hemorrhage (ARIA-H) is the second ARIA seen with anti-amyloid therapeutics. ARIA-H is described as sulcal or leptomeningeal hemosiderin deposits or parenchymal microhemorrhages seen on susceptibility weighted imaging or gradient echo sequence on MRI (Barakos et al., 2013; Arrighi et al., 2016) [Figures 1C,D reproduced from Sperling et al. (2011) with permission from Wiley] (Sperling et al., 2011).

The proposed pathophysiology of ARIA in the setting of anti-amyloid therapeutics is a result of inflammation causing increased leakiness of brain vessels (Withington and Turner, 2022; Hampel et al., 2023). The mobilization of the amyloid plaques in blood vessels of the brain is causes inflammation with resultant in swelling and microhemorrhages seen on MRI (Hampel et al., 2023). ARIA-E is thought to cause proteinaceous effusion or vasogenic edema. While the exact mechanism of ARIA-E is unknown, it is thought to be similar to cerebral amyloid angiopathy related inflammation (CAA-ri) (Martínez-Lizana et al., 2015; Yang et al., 2023), where perivascular inflammation due to amyloid or removal of amyloid plaque causes vasogenic edema that can cause MRI changes seen in CAA-ri or with association of anti-amyloid therapies (Martínez-Lizana et al., 2015; Antolini et al., 2021; Piazza et al., 2022). In fact, the amount of amyloid or amyloid burden can be associated with increased risk of ARIA (Klein et al., 2019).

Following anti-amyloid therapeutic administration, small microhemorrhages or hemosiderosis can form termed ARIA-H. ARIA-H is thought to develop in via similar mechanism to ARIA-E, development resulting from vascular leakiness of blood products into the brain parenchyma that results in small microhemorrhages or hemosiderosis (Salvarani et al., 2016; Hampel et al., 2023; Yang et al., 2023). Microhemorrhages, similar in appearance to ARIA-H are also seen with cerebral amyloid angiopathy (CAA) and are associated with increased age (Salvarani et al., 2016; Piazza et al., 2022).

## ARIA time course

The risk of developing either ARIA-H or ARIA-E is highest when first starting the anti-amyloid therapeutic (Filippi et al., 2022; Wang et al., 2022). ARIA-E incidence after initiation of bapineuzumab is highest between infusion two and three at 9% (Sperling et al., 2012). Incidence of ARIA-E decreases significantly after the second infusion of bapineuzumab with 0.7% of ARIA-E after the last infusion (5th) (Barakos et al., 2022). In the aducanumab trials the incidence of ARIA-E was highest in the first 6 months of infusions (Wang et al., 2022). The incidence of ARIA-H is also thought to decrease the longer a patient is on the anti-amyloid drug, but this is not as thoroughly reported (Barakos et al., 2022; Filippi et al., 2022).

The incidence of ARIA is variable with the different anti-amyloid therapeutics with ARIA-E incidence ranging from 0.9–40.6% (Table 1; Carlson et al., 2016; Avgerinos et al., 2021; Barakos et al., 2022; Jeong et al., 2022; Salloway et al., 2022; Vaz et al., 2022). The incidence of ARIA-H ranges from 0.5–28.4% (Barakos et al., 2022; Hampel et al., 2023). The variation in ARIA incidence may be due to the anti-amyloid antibody’s target. The highest incidence of ARIA has been in studies where the anti-amyloid therapeutic targeted the N terminus of the amyloid beta, while targets of the C terminus or mid-domain had lower incidences of ARIA (Avgerinos et al., 2021; Filippi et al., 2022; Yadollahikhales and Rojas, 2023). However, differences in population and study design may also contribute to differences in ARIA incidences.

**Table 1:** A table showing the incidence of ARIA-H and ARIA-E comparing Lecanemab (10 mg/kg), Aducanumab (10 m/kg), and Donanemab (700 mg for three infusions and then 1,400 mg).

	Lecanemab (1 Omg/kg)	Aducanumab (1 Omg/kg)	Donanemab (1,400 mg)
ARIA-H	17.3% (155)	Separated	31.4% (268)
Microhemorrhages	14.0% (126)	19% (197)	26.8% (229)
Superficial siderosis	5.6% (50)	14.7% (151)	15.7% (134)
ARIA-E (Total)	12.6% (113)	35.2% (362)	36.8% (314)
Symptomatic ARlA-E	2.8% (25)	9.1% (94)	6.1% (52)
Asymptomatic ARIA-E	9.7% (88)	26% (268)	17.9% (153)
ARIA-E separated by ApoE			
ApoE −/−	5.4% (15/278)	20.3% (72/355)	15.7% (40/255)
APOE −/+	15.8% (52/479)	43% (290/674)	22.8% (103/452)
APOE +/+	32.6% (46/141)	Included in carriers	40.6% (58/143)

ARIA-H is further separated by microhemorrhages and superficial siderosis. ARIA-E is separated into symptomatic vs. asymptomatic ARIA-E. Incidence of ARIA-E is then separated by apoE carrier status (−/− being a non carrier; +/− carrying one copy of the apoE ε4 allele; +/+ carrying two apoE ε4 alleles). Data is adapted from van Dyck et al. (2023), Salloway et al. (2022), and Sims et al. (2023).

ARIA-E and ARIA-H tend to be asymptomatic with most anti-amyloid therapies, however some ARIA can be severe, even leading to death (Sims et al., 2023). In the lecanemab trial of the 12.6% of treated patients developed ARIA-E and 22.1% of patient’s that developed ARIA-E had symptoms (van Dyck et al., 2023). Similarly with ARIA-H in the lecanemab trial 17.3% of treated patients developed ARIA-H and only 0.4% of patients that developed ARIA-H had symptoms (van Dyck et al., 2023). Symptoms with ARIA are typically headache, confusion, gait instability and vomiting (Barakos et al., 2022; Yadollahikhales and Rojas, 2023). However, severe reactions such as brain swelling, seizure and death have been reported with ARIA (Klein et al., 2022; Reish et al., 2023).

With discontinuation of the medication ARIA-E typically resolves within 6 months. However, some instances of ARIA-E have required short doses of corticosteroids, similar to the treatment of CAA-ri (Antolini et al., 2021; Filippi et al., 2022; Klein et al., 2022; Withington and Turner, 2022; Hampel et al., 2023). Recurrence of ARIA-E is seen in 25.6% of patients after resuming anti-amyloid therapy (Sperling et al., 2012; Ketter et al., 2017; Filippi et al., 2022). Even resuming anti-amyloid monoclonal antibody at a lower dose is associated with almost 15% development of ARIA-E relapse (Barakos et al., 2022; Filippi et al., 2022). ARIA-H unfortunately does not resolve with discontinuation of the drug and persists after identification on MRI (Arrighi et al., 2016; Klein et al., 2022).

## Dose dependent

A dose dependent relationship with increased doses of anti-amyloid therapeutics and ARIA has been seen with most of the anti-amyloid monoclonal antibodies that have gone to clinical trials. In the two phase 3 bapineuzumab studies; ARIA-E was seen in 5.6% of 0.5 mg/kg group, 13.4% of 1 mg/kg group and 19.9% of 2 mg/kg group (Sperling et al., 2012; Barakos et al., 2013). In a phase 3 aducanumab study participants given 6 mg/kg had a 21.2% incidence while the 10 mg/kg group had a 35.2% incidence of ARIA-E (Barakos et al., 2022; Budd Haeberlein et al., 2022; Filippi et al., 2022; Vaz et al., 2022). Similarly lecanemab patients that received 10 mg/kg had a 9.9% ARIA-E incidence while the 5 mg/kg group had a 3.3% incidence and 2.5 mg/kg group had a 1.9% incidence of ARIA-E (Honig et al., 2023; van Dyck et al., 2023).

Dose dependent incidence of ARIA-H is not as evident. In bapineuzumab studies ARIA-H incidence is 24.9 and 28 and 28.4% in the bapineuzumab doses 0.5 mg/kg, 1 mg/kg and 2 mg/kg, respectively (Barakos et al., 2013; Arrighi et al., 2016; Roytman et al., 2023). In the aducanumab studies ARIA-H (microhemorrhages) was seen in 16% of the patients treated with the low dose and 20% in the patient’s treated in the high dose (Budd Haeberlein et al., 2022; Cummings et al., 2022). Incidence of ARIA-H is associated with ARIA-E, where the patients that have ARIA-E have higher incidence of ARIA-H (Barakos et al., 2022; Filippi et al., 2022). In the lecanemab study, 17.3% of all treated patients developed ARIA-H, however 48.4% of patients that developed ARIA-H also developed concurrent ARIA-E (Cummings et al., 2023; van Dyck et al., 2023).

## ApoE ε4

Patients that carry the apoE ε4 allele have higher risk for developing Alzheimer’s disease than non-carriers (Raulin et al., 2022; Lou et al., 2023). Patients that carry the apoE ε4 allele also have a higher risk of leaky vessels or blood brain barrier permeability, likely leading to increased microhemorrhages or edema (Montagne et al., 2020; Moon et al., 2021; Chen et al., 2023). The apoE ε4 allele is associated with increased cerebral amyloid deposition in blood vessel walls (Antolini et al., 2021). Pretreatment, patients with this allele have higher risks of CAA and cerebral microhemorrhages (Ulrich et al., 2018; Piazza et al., 2022).

Carriers of the apoE ε4 allele has been shown to correlate with increased blood brain barrier permeability (Montagne et al., 2020; Moon et al., 2021; Chen et al., 2023), likely this is related to increased risk of ARIA seen with apoE ε4 allele carriers in anti-amyloid therapeutic trials (Barakos et al., 2022). Homozygosity of the apoE allele also seems to increase incidence of ARIA-E and ARIA-H (Salloway et al., 2022; Sims et al., 2023; van Dyck et al., 2023). In the bapineuzumab studies patients with one copy of the apoE ε4 allele (heterozygotes) had a hazard ratio of 4.10 in developing ARIA-H and those with two apoE ε4 alleles (homozygotes) had a hazard ratio of 12.79 (Barakos et al., 2013; Arrighi et al., 2016; Filippi et al., 2022). In lecanemab studies 39% of people with two apoE ε4 alleles developed ARIA-H compared to 19.7% of apoE ε4 allele carriers, with only 11.9% of noncarriers developed ARIA-H (van Dyck et al., 2023).

A similar association was seen between ARIA-E and apoE ε4 allele carriers (one or two alleles). In studies with gantenerumab, donanemab, aducanumab, and lecanemab patients with the apoE ε4 allele have increased association with developing ARIA-E (Budd Haeberlein et al., 2022; Wang et al., 2022; Withington and Turner, 2022; Qiao et al., 2023; Roytman et al., 2023; Sims et al., 2023; van Dyck et al., 2023). In the gantenerumab studies, apoE ε4 carriers were 5 times more likely to develop ARIA-E than noncarriers (Joseph-Mathurin et al., 2022). In the aducanumab study, patients that were in the 10 mg/kg treated group that were apoE ε4 carriers had an ARIA-E incidence of 43% vs. an incidence of 18% in noncarriers (Barakos et al., 2022; Withington and Turner, 2022; Hampel et al., 2023). Again, homozygosity of ApoE ε4 allele in the aducanumab study was associated with significantly increased hazard ratio of developing ARIA-H compared to heterozygotes and noncarriers (Budd Haeberlein et al., 2022; Cummings et al., 2022; Salloway et al., 2022). Of the 21/25 participants in the lecanemab study that had symptomatic ARIA-E were apoE ε4 carriers (van Dyck et al., 2023). In lecanemab studies 15.4% of apoE ε4 heterozygotes developed ARIA-E while only 5.4% of noncarriers developed ARIA-E (Honig et al., 2023; Qiao et al., 2023; van Dyck et al., 2023). Similarly 40% of apoE ε4 homozygotes had ARIA-E, while 22.8% of apoE ε4 heterozygotes developed ARIA-E (Sims et al., 2023).

Case reports suggest that while symptomatic ARIA is relatively uncommon, apoE ε4 homozygotes are more likely to be symptomatic and have severe clinical manifestations requiring corticosteroids. A participant who had apoE ε4 homozygosity treated with aducanumab developed severe ARIA-E with headaches, encephalopathy and alexia requiring treatment with corticosteroids (Filippi et al., 2022); while another participant homozygous with apoE ε4 treated with aducanumab had severe ARIA-E developing encephalopathy, epileptiform discharges, malignant hypertension and required corticosteroids (Vande Vrede et al., 2020). While symptomatic ARIA is rare it is more common in apoE ε4 carriers, especially those that are homozygous ARIA is more likely to be symptomatic and severe (Vande Vrede et al., 2020; Barakos et al., 2022; Honig et al., 2023).

## Cerebral microhemorrhages

Existing cerebral microhemorrhages are also a risk factor for developing significant ARIA-E and ARIA-H. Early studies such as those with pre-treatment cerebral microhemorrhages treated with gantenerumab showed an odds ratio of 13.7 for developing ARIA-E than those without cerebral microhemorrhages (Joseph-Mathurin et al., 2022; Wang et al., 2022). Patients that have cerebral microhemorrhages pre-treatment were 1.7 times more likely to develop ARIA-E than those that did not have microhemorrhages in the aducanumab trial (Budd Haeberlein et al., 2022; Vaz et al., 2022). In bapineuzumab trials, participants with cerebral microhemorrhages at baseline had a hazard ratio of 3.58 in developing ARIA-H (Sperling et al., 2012; Arrighi et al., 2016). Participants with more than four microhemorrhages were excluded from the aducanumab and lecanemab trials due to the risk for increased ARIA-E and ARIA-H risk (Cummings et al., 2022).

## Antithrombotic use

Antithrombotic use is a significant risk factor for developing ARIA-H. In the bapineuzumab trials antithrombotic use was associated with a hazard ratio of 2.20 for developing ARIA-H. However, in the aducanumab studies there was no increased risk of developing ARIA-H with concomitant aspirin and anticoagulation use. However, in the recommendations for aducanumab it was recommended that in patients that develop conditions that require anti-coagulation such as atrial fibrillation, deep vein thrombosis, or a pulmonary embolism should stop the anti-amyloid therapy (Cummings et al., 2022). In the lecanemab trial participants that received aspirin up to 325 mg daily or clopidogrel up to 75 mg were allowed to continue in the trial (Cummings et al., 2023). However, in the lecanemab trial a participant who received tissue plasminogen activator for acute ischemic stroke developed many microhemorrhages leading to death (Reish et al., 2023). Use of tissue plasminogen activator is contraindicated in patients receiving anti-amyloid treatment especially lecanemab (Cummings et al., 2023).

## Age

Age is a risk factor for increased microbleeds and CAA which may contribute to increased risk of developing ARIA-H. Co-morbid CAA is thought to be a risk factor for developing ARIA (Sveikata et al., 2022). Increased amyloid burden has been shown to have increased risk of ARIA (Klein et al., 2019; Barakos et al., 2022). In the aducanumab studies there was a slightly increased hazard ratio for developing ARIA-H (1.06) with increased age while there was no association between age and ARIA-E. In the gantenerumab study, age was not associated with increased risk of ARIA, however severity of ARIA-E was associated with increased age (Joseph-Mathurin et al., 2022). Age of onset of symptoms should be something to consider as younger patients are more likely to be associated with apoE ε4 allele. The lecanemab study included a wide range of patients from 50–90 years of age, likely including both late and early onset Alzheimer’s (van Dyck et al., 2023). It is possible that early-onset and late-onset Alzheimer’s have different risks and rates of developing ARIA. Determining the amyloid burden prior to treatment is likely to play an important role in determining initiation of anti-amyloid treatment (Klein et al., 2019; Barakos et al., 2022).

## Prior strokes

Participants with prior ischemic and hemorrhagic strokes involving the basal ganglia or large areas of vascular territory were excluded in the lecanemab study (Cummings et al., 2023; van Dyck et al., 2023). Participants with prior hemorrhagic or ischemic strokes have been excluded from anti-amyloid therapeutic trials starting with gantenerumab (Joseph-Mathurin et al., 2022; Wang et al., 2022). The presence of more than two lacunar strokes or a stroke in a major vascular territory were part of the exclusion criteria for lecanemab as strokes have been associated with increased vascular leakiness which could lead to increased risk for ARIA-H or ARIA-E (Cummings et al., 2023). For instance, ischemic stroke like symptoms were seen in a patient treated with donamemab who developed a quadrantanopsia with FLAIR changes on MRI, thought to be CAA-ri vs. ARIA-E that resolved 4 weeks later with steroid treatment (Kmeid et al., 2023). In the lecanemab study, a patient developed stroke like symptoms resulting in tPA administration that resulted in hemorrhagic strokes leading to death (Reish et al., 2023).

## Other considerations

While trials have been conducted on anti-amyloid therapeutics for decades, the cumulative effect of years of anti-amyloid monoclonal antibodies on patients is yet to be determined. Patients who have received aducanumab or lecanemab commercially have only received it for months to 18 months (Yadollahikhales and Rojas, 2023). The long-term effects or other adverse effects have yet to be identified. Whether there is benefit past 18 months with lecanemab remains to be seen. As age has been associated with higher incidence of ARIA, as patients age and Alzheimer’s disease naturally progresses, there is a greater risk for developing ARIA or symptomatic ARIA (Mo et al., 2017; Vaz et al., 2022). It is possible that the risk of ARIA over time, especially in those that are treated over a long period of time may be cumulative and may be higher than previously reported. However, it is also possible that early treatment may reduce the cumulative risk of ARIA, as ARIA risk is highest after initiation of the anti-amyloid therapeutic (Barakos et al., 2022; Salloway et al., 2022). The full implications of ARIA and long term anti-amyloid therapeutic use has not been investigated and is an important area of future research.

While cerebral microbleeds are a risk factor for ARIA it is unknown whether other vascular changes such as enlarged perivascular spaces are a risk factor for ARIA (Salloway et al., 2022). Enlarged perivascular spaces are thought to be a risk factor for CAA and Alzheimer’s (Foley et al., 2023; Jia et al., 2023; Voorter et al., 2023; Zebarth et al., 2023). Further research is needed to determine if enlarged perivascular spaces pre-treatment are a risk factor for ARIA. Further research would also be needed to evaluate if perivascular spaces can monitor for risk of developing ARIA-H or ARIA-E.

Race/ethnicity is also an important consideration as most participants that were studied in the initial clinical trials for aducanumab and lecanemab were Caucasian (Mo et al., 2017; Budd Haeberlein et al., 2022; van Dyck et al., 2023). How these medications may work in Black, Asian, or Hispanics etc. may be very different than what has currently been reported. The sample size in other races/ethnicities besides Caucasian were too small to adequately assess the incidence of ARIA-H and ARIA-E in these races/ethnicities with mentioned risk factors.

Sex differences are also a consideration with further use of these anti-amyloid therapeutics. While there have been no differences in side effects with ARIA in the initial trials for anti-amyloid therapeutics (Barakos et al., 2022; Filippi et al., 2022), it is possible that this could develop as some sex differences have been seen in efficacy of these therapeutics. For instance, in the lecanemab trial there was a 27% delay in progression but when split between men and women, 43% of men had a delay in progression however only 12% of women had a delay in progression (van Dyck et al., 2023).

Sex differences are seen in patients with Alzheimer’s disease who are carriers of ApoE ε4 allele. Women who have Alzheimer’s and have apoE ε4 allele are shown to have worsened Alzheimer’s symptoms than men who also have Alzheimer’s and apoE ε4 allele (Pan et al., 2023; Walters et al., 2023). While sex differences and apoe ε4 allele carrier status were investigated in anti-amyloid therapeutic trial the interaction of apoE ε4 allele and sex differences was not specifically evaluated. Further research of the interaction of Alzheimer’s and apoE ε4 allele carrier status in patients treated with anti-amyloid therapeutics is needed.

While currently there has been no association with hypertension and development of ARIA, it is possible that participants in the trials were more adherent to their medications than the general public and that the sample size of the trials were too small (Barakos et al., 2022; Budd Haeberlein et al., 2022; Filippi et al., 2022). Though a small sample size 30% of patients with history of hypertension treated with gantenerumab developed ARIA-E that only 11.9% of patients without hypertension developed ARIA-E (Joseph-Mathurin et al., 2022). Given that the proposed pathophysiology of ARIA-E and ARIA-H is thought to be due to vascular injury or vascular leakiness, one would expect that uncontrolled hypertension or other vascular risk factors like diabetes mellitus or smoking would associate with increased incidence of ARIA-H and ARIA-E (Barakos et al., 2013).

## Discussion

Anti-amyloid monoclonal antibodies have inspired a lot of hope in the Alzheimer’s disease community. However, risk factors for the development of ARIA should be assessed and mitigated prior to starting anti-amyloid therapy. Risk factors to consider include age, prior stroke and cerebral microhemorrhages, antithrombotic/anticoagulant use, apoE ε4 allele carrier status, and dose of the drug. Many Alzheimer’s disease patients have many of these risk factors prior to initiation of anti-amyloid therapeutics. Thus, patient selection for anti-amyloid therapy should be very rigorous and patients should understand the risks and limited benefit they may see from starting such medications. Ultimately, it is likely that there will be a very small population of Alzheimer’s disease patients that have minimal risk factors and are able to start these anti-amyloid therapies. Even in people who can get anti-amyloid therapeutics, administration every two weeks may not be feasible for 5, 10 or 15 consecutive years. While cumulative ARIA risk and effects of long-term anti-amyloid therapy use is unknown, rigorous monitoring and research is needed to fully evaluate safety and efficacy of these new anti-amyloid therapeutics. Additional risk factors such as long-term effects, race/ethnicity, sex, perivascular spaces, and additional vascular risk factors need further research with use of anti-amyloid monoclonal antibodies.

Unfortunately, many memory clinic patients have the risk factors for ARIA including age, apoE ε4, amyloid burden, anti-thrombotic use, prior strokes, and prior hemorrhages that would preclude patient’s from getting these anti-amyloid therapeutics. Given the FDA’s guidance on exclusion and inclusion criteria for administration of anti-amyloid therapeutics it has been estimated that only 5% of patients would be eligible for aducanumab and 8–13% of patients would be eligible for lecanemab (Rosenberg et al., 2022; Pittock et al., 2023). While these new therapeutics are exciting and may help some patients, more work must be done to help the wider population of Alzheimer’s patients.

## Author contributions

SD: Writing – original draft. RS: Writing – review & editing.

## Supplementary material

Supplementary Table S1 Inclusion and exclusion criteria for receiving lecanemab infusions adapted from Cummings et al. (2023).
