# Supplementary figures and images for: Risk factors in developing amyloid related imaging abnormalities (ARIA) and clinical implications
Source: Front Neurosci. 2024 Jan 19;18:1326784. doi: 10.3389/fnins.2024.1326784 (PMC10834650; doi:10.3389/fnins.2024.1326784)

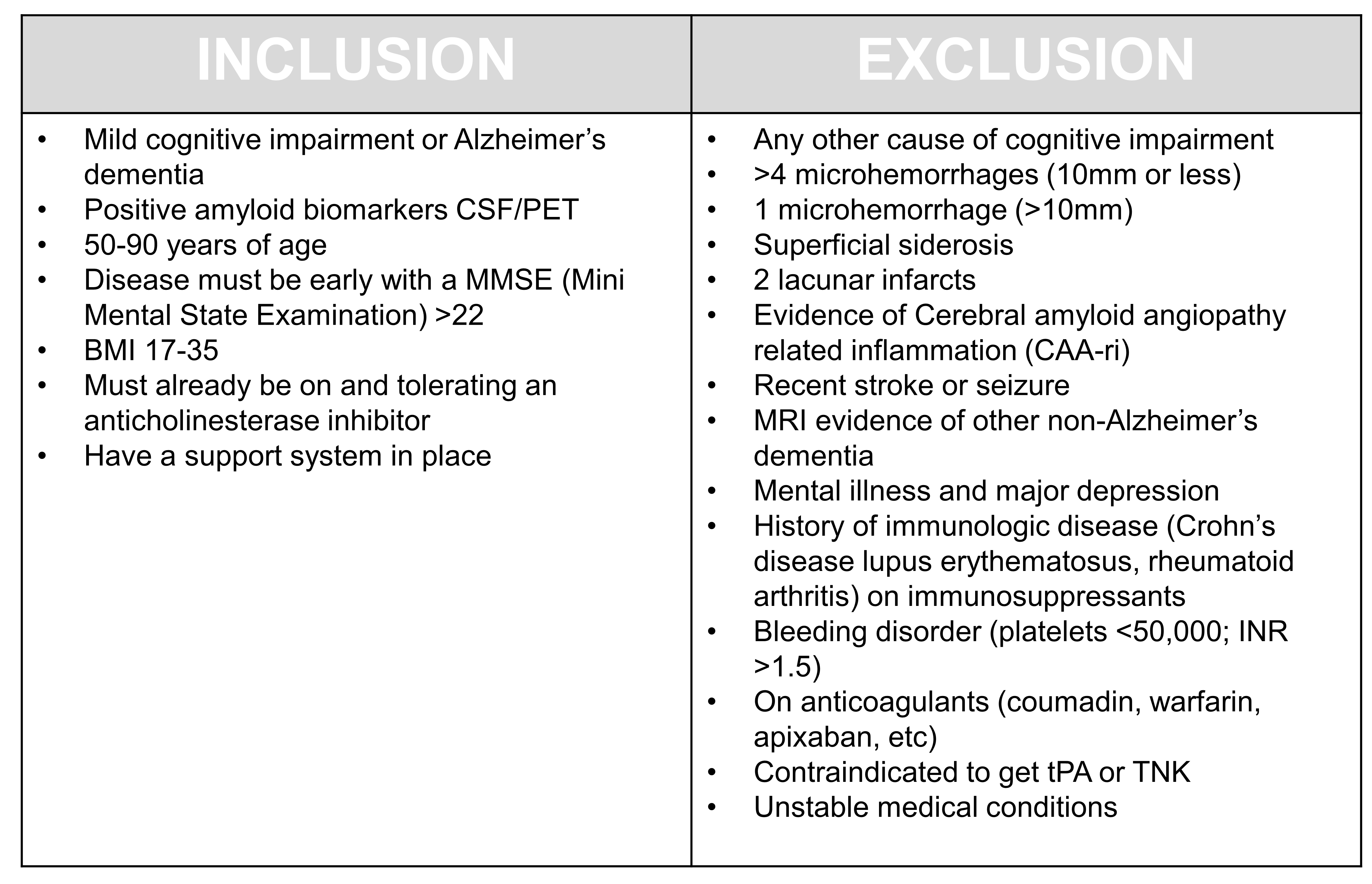

Supplement: Supplementary Table S1 — Inclusion and exclusion criteria for receiving lecanemab infusions adapted from Cummings et al. (2023). [file Image_1.TIF]
